# Neighborhood Deprivation and Biological and Psychosocial Outcomes for Head and Neck Cancer

**DOI:** 10.1001/jamanetworkopen.2025.38569

**Published:** 2025-10-21

**Authors:** Canhua Xiao, Andrew H. Miller, Sudeshna Paul, Veronika Fedirko, Gang Peng, Karen N. Conneely, Jennifer C. Felger, Dong M. Shin, Nabil F. Saba, Deborah W. Bruner

**Affiliations:** 1 Emory University School of Nursing, Atlanta, Georgia; 2 Emory University School of Medicine, Atlanta, Georgia; 3 Department of Epidemiology, MD Anderson Cancer Center, Houston, Texas]; 4 Department of Medical and Molecular Genetics, Indiana University School of Medicine, Indianapolis

## Abstract

This cohort study examines the association of neighborhood disadvantage with outcomes in patients with head and neck cancer.

## Introduction

The critical role of social determinants of health (SDOH) on disease outcomes has been increasingly recognized.^1^ However, the effects of SDOH at the neighborhood level on biological and psychosocial outcomes are not well-documented in cancer, particularly head and neck cancer (HNC). The purpose of this study was to examine the associations of neighborhood disadvantages with HNC outcomes.

## Methods

This cohort study was approved by the Emory University institutional review board. All participants provided informed consent. This study is reported following the Strengthening the Reporting of Observational Studies in Epidemiology (STROBE) reporting guideline. This longitudinal study recruited patients with newly diagnosed HNC; data were collected before, at the end of, and 1 year after radiotherapy.

Race was identified through electronic health records (EHRs). SDOH data were collected at baseline using the national Area Deprivation Index (ADI) rank to assess neighborhood disadvantage.^2^ Tumor human papillomavirus (HPV) status was determined through EHR review. Whole blood was collected for assays of DNA methylation, inflammatory markers, and short-chain fatty acids (SCFAs). DNA methylation was used to calculate epigenetic age acceleration, including DunedinPACE, GrimAge, and PhenoAge.^3^ Plasma inflammation markers included C-reactive protein (CRP) and interleukin 6 (IL-6), and plasma SCFAs included butyrate, propionate, and acetate. Psychosocial outcomes included perceived stress, depressive symptoms, sleep, fatigue, cognition, and pain.

Logistic and linear regression were performed to examine whether ADI was associated with HPV-associated tumors and SCFAs, respectively, before radiotherapy. Generalized estimating equations were conducted to assess whether ADI was associated with biological and psychosocial outcomes over time. We also examined whether ADI moderated the association between HPV status and the biological and psychosocial outcomes. *P* values were 2-sided, and statistical significance was set at 0.05, unless otherwise indicated. Data were analyzed using SPSS version 21 (IBMS)and R 4.5.1(R Project for Statistical Computing ) from December 2024 to August 2025 August. Details are in the eMethods in Supplement 1.

## Results

The study enrolled 193 patients (Table). Patients with HNC living in more deprived areas were more likely to have HPV-unassociated tumors, while those residing in less deprived areas were more likely to have HPV-associated tumors ( B [SE] = −0.016 [0.007]; *P* = .03). Every 1-unit increase in ADI was associated with a 2% increase in the odds of being diagnosed with HPV-unassociated tumors (odds ratio, 1.02; 95% CI, 1.00 to 1.03; *P* = .03). Neighborhood deprivation was further associated with accelerated epigenetic age (DunedinPACE: B [SE] = 0.001 [0.0004]; *P* < .001; GrimAge: B [SE] = 0.067 [0.013]; *P* < .001), heightened inflammation (log[CRP]: B [SE] = 0.004 [0.002]; *P* = .005), decreased propionate (B [SE] = −0.001 [0.001]; *P* = .02), increased stress (B [SE] = 0.055 [0.020]; *P* = .005), and lowered sleep quality (B [SE] = 0.022 [0.011]; *P* = .04) (Figure). All analyses were adjusted for covariates and remained significant after Bonferroni correction.

**Table.** Baseline Demographic and Clinical Characteristics of the Participants

Characteristics	Patients, No. (%) (N = 193)
Age, mean (SD), y	59.49 (10.08)
Sex	
Male	145 (75.1)
Female	48 (24.9)
Race^a^	
Black	30 (15.7)
White	155 (80.3)
Other	8 (4.0)
Marital status^b^	
Married	133 (70.0)
Unmarried	57 (30.0)
Total with data, No.	190
History of tobacco use	
No	75 (39.5)
Yes	115 (60.5)
Total with data, No.	190
History of alcohol use	
No	102 (54.3)
Yes	86 (45.7)
Total with data, No.	188
BMI	
Mean (SD)	27.87 (5.66)
Total with data, No.	191
Comorbidities^c^	
0	118 (61.1)
1	44 (22.8)
≥2	31 (16.1)
Antidepressants	
No	153 (81.0)
Yes	36 (19.0)
Total with data, No.	189
Cancer site	
Oropharynx	101 (52.3)
Other	92 (47.7)
Stage	
≤III	46 (24.0)
IV	146 (76.0)
Total with data, No.	192
HPV status	
HPV associated	97 (50.3)
HPV unassociated	96 (49.7)
Treatment	
RT	11 (5.7)
RT and surgery	31 (16.1)
Chemotherapy and RT	103 (53.4)
Chemotherapy, RT, and surgery	48 (24.9)
Chemotherapy	
Cisplatin	98 (64.9)
Carboplatin/paclitaxel	32 (21.2)
Other	21 (13.9)
Radiation dose, mean (SD), Gy	66.24 (7.16)
Feeding tubes	
No	79 (42.7)
Yes	106 (57.3)
Total with data, No.	185

Abbreviations: BMI, body mass index (calculated as weight in kilograms divided by height in meters squared); HPV, human papillomavirus; RT, radiation therapy.

^a^ Race was assessed according to the protocol. Other included Native American and unknown.

^b^ Married included patients married or living as married; unmarried included patients single, separated, divorced, or widowed.

^c^ Comorbidities were assessed using the Charlson Comorbidity Index, excluding tumor.

**Figure.**
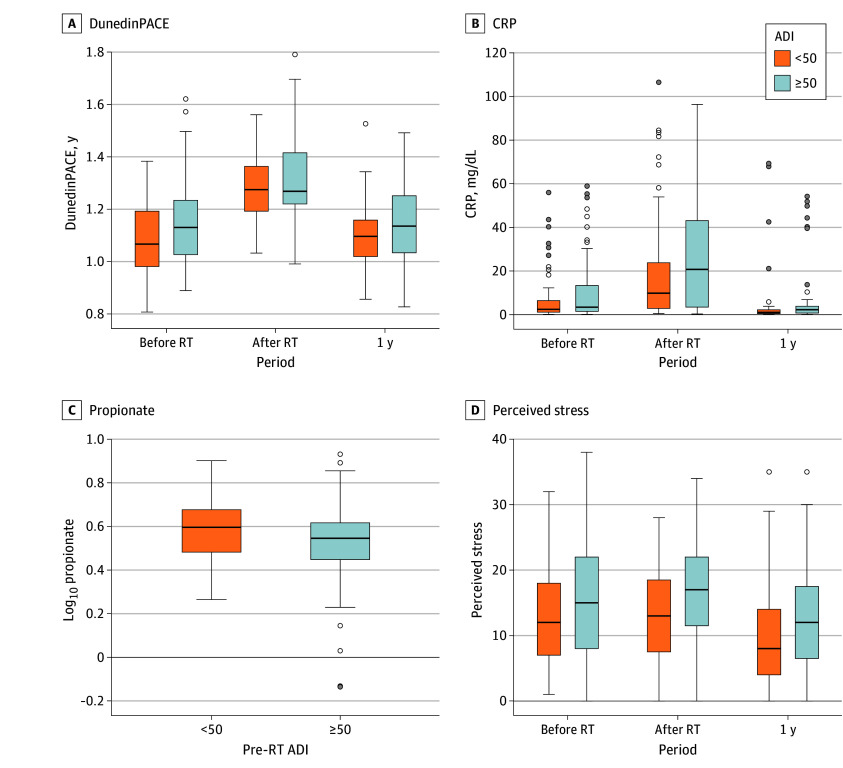
Associations Between ADI and DunedinPACE, CRP, Propionate, and Perceived Stress The line within the box represents the median, with the upper and lower edges of the box representing the 25th and 75th percentiles and the whiskers representing the range. Dots indicate outliers. ADI indicates Area Deprivation Index; CRP, C-reactive protein (to convert to milligrams per liter, multiply by 10); RT, radiotherapy.

Interaction analyses showed significant moderating effects of ADI on the associations between IL-6 (B = 0.118 [0.038]; *P* = .002), as well as butyrate (B = 5.391 [2.613]; *P* = .04), and HPV status. In more deprived neighborhoods, heightened inflammation (IL-6: B [SE] = 0.182 × 10^−3^ [0.088 × 10^−3^]; *P* = .04) and decreased SCFAs (butyrate: B [SE] = −7.130 [2.996]; *P* = .02) were associated with increased likelihood of HPV-unassociated tumors versus HPV-associated tumors, while the association was not significant in less deprived neighborhoods (IL-6: B [SE] = −0.018 [0.030]; *P* = .56; butyrate: B [SE] = 0.896 [1.450]; *P* = .54).

## Discussion

The findings of this cohort study suggest a pivotal role of neighborhood disadvantage on multiple adverse HNC outcomes, including HPV-unassociated tumors, accelerated epigenetic aging, heightened inflammation, decreased beneficial gut metabolites (SCFAs), and distressed psychological status. All these outcomes have been associated with increased morbidity and mortality,^4,5,6^ further underscoring the significant health impact of SDOH. Our results also highlight that socioeconomic inequities complicate HNC control and prevention. In poorer neighborhoods, health efforts should focus on HPV vaccination as well as managing traditional types of HNC. In less disadvantaged areas, HPV vaccination is still crucial. Other public health initiatives, such as food insecurity, lack of insurance, and poor access to health care, should be considered to reduce the vulnerability of patients from disadvantaged neighborhoods to adverse cancer outcomes.

These findings should be interpreted with caution, as other factors, such as race, diet, and physical activity, may contribute to adverse outcomes. Our findings are limited to patients with HNC and from a single institute

## Notes

Supplement 1.
eMethods.

Supplement 2.
Data Sharing Statement
