# Error in the Figure

**DOI:** 10.1001/jamanetworkopen.2025.49575

**Published:** 2025-11-24

In the Research Letter titled “Neighborhood Deprivation and Biological and Psychosocial Outcomes for Head and Neck Cancer,”^1^ published October 21, 2025, there was an error in panel C of the Figure. This article has been corrected.^1^
