# Supplementary material for: Neighborhood Deprivation and Biological and Psychosocial Outcomes for Head and Neck Cancer
Source: JAMA Netw Open. 2025 Oct 21;8(10):e2538569. doi: 10.1001/jamanetworkopen.2025.38569 (PMC12541531; doi:10.1001/jamanetworkopen.2025.38569)
Supplement: Supplement 1. — eMethods. [file jamanetwopen-e2538569-s001.pdf]

Supplemental Online Content

Xiao C, Miller AH, Paul S, et al. Neighborhood deprivation and biological and psychosocial outcomes for head and neck cancer. *JAMA Netw Open*. 2025;8(10):e2538569. doi:10.1001/jamanetworkopen.2025.38569

eMethods.

This supplemental material has been provided by the authors to give readers additional information about their work.

© 2025 Xiao C et al. *JAMA Network Open*.

eMethods:

This was a longitudinal study with data collected prior to radiotherapy, immediately at the end of radiotherapy, and one year post-radiotherapy. Radiotherapy is often six to seven weeks with or without concurrent chemotherapy. Surgery, if needed, usually occurs one month before radiotherapy. The study was approved by the Emory University Institutional Review Board; all study participants provided informed consent.

Exposure:

Social determinants of health data were collected at baseline and focused on neighborhood measures, a key component for social determinants of health according to the World Health Organization (WHO) framework.[1] We used the **area deprivation index (ADI)**, based on a measure created by the Health Resources & Services Administration, to assess neighborhood disadvantage. The ADI is a reliable measure that reflects a geographic area's level of socioeconomic deprivation and is associated with health outcomes.[2] The ADI accounts for income, education, employment, and housing quality at the neighborhood level or each census block group. The ADI national score is a percentile ranking ranging from 1 to 100, with higher scores indicating more disadvantaged or deprived neighborhoods. Patients' address data at baseline were assigned an ADI national rank according to their residential census block group.[3]

Health Outcomes:

Tumor human papillomavirus (HPV) status was determined through chart review, with P16-positive cases classified as HPV-associated tumors. P16-negative cases or those lacking HPV

© 2025 Xiao C et al. *JAMA Network Open*.

testing (indicating a low likelihood of HPV association) were categorized as non-HPV-associated tumors.

<u>*Biological outcomes:*</u> Whole blood was collected into chilled EDTA tubes to isolate plasma and leukocytes (buffy coat) using standardized protocols[4,5] and stored at -80ºC until batched assays for DNA methylation, inflammatory markers, and short chain fatty acids (SCFAs).

Epigenetic age acceleration: DNA was extracted using the QIAamp DNA mini kit (Qiagen), and DNA methylation levels were quantified using MethylationEPIC BeadChip (Illumina, San Diego, CA) following manufacturers' protocols at the Emory Integrated Genomics Core. The β values of DNAm levels were corrected using quantile normalization techniques[6] followed by standard quality control,[7] leading to the removal of 59134 out of 866238 probes. All samples passed the sample level of quality control: excluding those with a median of methylation and unmethylation signal <10.5,[6] outliers in principal component analysis and clustering, and those with low-quality probes >1%.

Epigenetic age acceleration (EAA) was calculated for six commonly used epigenetic clocks: DunedinPACE, GrimAge, PhenoAge, Hannum, Horvath 2013, and Horvath 2018,[8-12] as they use different CpG sites and may capture different molecular processes in aging.[13] With the exception of DunedinPACE, EAA was further estimated as the residual resulting from regressing epigenetic age on chronological age.[14] A higher value in EAA estimate indicates accelerated aging. DNA methylation data were available at baseline, at the end of radiotherapy, and twelve months post-radiotherapy.

Inflammatory markers: Plasma concentrations of interleukin 1 receptor antagonist (IL1ra), IL1β, IL6, IL10, tumor necrosis factor α (TNFα), soluble TNF receptor 2 (sTNFR2),

© 2025 Xiao C et al. *JAMA Network Open*.

were determined in duplicate using multiplex assays (R&D Systems, Minneapolis, MN). C-reactive protein (CRP) was measured using a standard turbidimetric assay.[4] All samples were assayed in duplicate, and the inter- and intra-assay coefficients of variation were reliably less than 10%. No values were below the limits of assay detection. Inflammatory marker data were available at baseline, end of radiotherapy, and twelve months post-radiotherapy.

Short-chain fatty acids: The Emory Integrated Lipidomics Core performed assays for free fatty acids (FFAs) in plasma. Based on the literature,[15-19] the FFA panel included three major SCFAs: butyrate, acetate, and propionate. The concentration of each detected FFA was determined based on five-point calibration with external standards within the range of 0.1 nM to 100 uM.

Psychosocial outcomes included perceived stress, depressive symptoms, sleep problems, fatigue, cognitive function, and pain. Perceived stress was measured using the Perceived Stress Scale (PSS).[20] Depressive symptoms was measured using the eight-item Patient Health Questionnaire (PHQ-8).[21,22] Sleep problems were measured with the Pittsburgh Sleep Quality Index (PSQI).[23-25] The Multidimensional Fatigue Inventory (MFI)-20 was used to measure fatigue. [26] The Patient-Reported Outcomes version of the Common Terminology Criteria for Adverse Events (PRO-CTCAE) was used to measure cognitive function and pain.[27] All these questionnaires have established reliability and validity and have been used in cancer populations.[28] Higher scores indicate severer symptoms.

Covariates:

© 2025 Xiao C et al. *JAMA Network Open*.

Demographic and clinical data were gathered using standard questionnaires and chart reviews. Demographic information included age, sex, race, marital status, tobacco use history (defined as cigarette smoking for at least one year), and alcohol use history (defined as consuming one or more drinks per week in the past year). Clinical data included body mass index (BMI), comorbid conditions (measured by the Charlson Comorbidity Index), primary cancer site, cancer stage (TNM), surgical history, chemotherapy, and tumor HPV status.

Statistical analyses:

Logistic regression was performed to examine whether ADI was predictive of HPV-associated head and neck cancer (HNC) diagnoses (yes vs. no) prior to radiotherapy. Covariates in the final models were selected based on the bivariate associations with HPV-associated HNC diagnoses and at a prior. Generalized estimating equations (GEE) were conducted to assess whether ADI was predictive of biological outcomes (i.e., epigenetic age acceleration and inflammation) and psychosocial outcomes (i.e., perceived stress, sleep problems, fatigue, depressive symptoms, cognitive functions, and pain) over time. Given the limit of only having SCFA data at baseline, linear regression models were performed to assess whether ADI was predictive of SCFAs prior to radiotherapy. Additionally, our early data demonstrated that patients with HPV-unassociated HNC experienced more negative biological responses and worse psychosocial outcomes compared to those with HPV-associated HNC.[29,30] Thus, we further examined whether ADI may moderate the association between HPV status and the biological and psychosocial outcomes. Bonferroni correction was employed for epigenetic age (p=0.05/6=0.008), inflammatory markers (p=0.05/7=0.007), SCFA (p=0.05/3=0.017, and psychosocial outcomes (0.05/6=0.008). For the moderation analysis, patients were stratified as having low (<50) or high (>=50) ADI based on

© 2025 Xiao C et al. *JAMA Network Open*.

the national median ADI. SPSS version 21 and R were used for data analyses.

References

1. WHO. A conceptual framework for action on the social determinants. World Health Organization. https://www.who.int/publications/i/item/9789241500852. Published 2010. Accessed2021.
2. Kind AJH, W B. Making Neighborhood Disadvantage Metrics Accessible: The Neighborhood Atlas. *New England journal of medicine.* 2018;378(26):2456-2458.
3. 2021Area Deprivation Index v4.01. 2024. https://www.neighborhoodatlas.medicine.wisc.edu/. Accessed 2023.
4. Xiao C, Beitler JJ, Higgins KA, et al. Fatigue is associated with inflammation in patients with head and neck cancer before and after intensity-modulated radiation therapy. *Brain Behav Immun.* 2016;52:145-152.
5. Xiao C, Beitler JJ, Higgins KA, et al. Differential regulation of NF-kB and IRF target genes as they relate to fatigue in patients with head and neck cancer. *Brain Behav Immun.* 2018;74:291-295.
6. Fortin JP, Triche TJ, Jr., Hansen KD. Preprocessing, normalization and integration of the Illumina HumanMethylationEPIC array with minfi. *Bioinformatics (Oxford, England).* 2017;33(4):558-560.
7. Chen Y-a, Lemire M, Choufani S, et al. Discovery of cross-reactive probes and polymorphic CpGs in the Illumina Infinium HumanMethylation450 microarray. *Epigenetics : official journal of the DNA Methylation Society.* 2013;8(2):203-209.
8. Levine ME, Lu AT, Quach A, et al. An epigenetic biomarker of aging for lifespan and healthspan. *Aging.* 2018;10(4):573-591.
9. Horvath S. DNA methylation age of human tissues and cell types. *Genome biology.* 2013;14(10):R115.
10. Horvath S, Oshima J, Martin GM, et al. Epigenetic clock for skin and blood cells applied to Hutchinson Gilford Progeria Syndrome and ex vivo studies. *Aging.* 2018;10(7):1758-1775.
11. Hannum G, Guinney J, Zhao L, et al. Genome-wide methylation profiles reveal quantitative views of human aging rates. *Mol Cell.* 2013;49(2):359-367.
12. Lu AT, Quach A, Wilson JG, et al. DNA methylation GrimAge strongly predicts lifespan and healthspan. *Aging (Albany NY).* 2019;11(2):303-327.
13. Zhao W, Ammous F, Ratliff S, et al. Education and Lifestyle Factors Are Associated with DNA Methylation Clocks in Older African Americans. *Int J Environ Res Public Health.* 2019;16(17).

© 2025 Xiao C et al. *JAMA Network Open*.

14. Levine ME, Hosgood HD, Chen B, Absher D, Assimes T, Horvath S. DNA methylation age of blood predicts future onset of lung cancer in the women's health initiative. *Aging.* 2015;7(9):690-700.
15. den Besten G, van Eunen K, Groen AK, Venema K, Reijngoud D-J, Bakker BM. The role of short-chain fatty acids in the interplay between diet, gut microbiota, and host energy metabolism. *Journal of lipid research.* 2013;54(9):2325-2340.
16. Sowah SA, Hirche F, Milanese A, et al. Changes in Plasma Short-Chain Fatty Acid Levels after Dietary Weight Loss Among Overweight and Obese Adults over 50 Weeks. *Nutrients.* 2020;12(2):452.
17. Nishitsuji K, Xiao J, Nagatomo R, et al. Analysis of the gut microbiome and plasma short-chain fatty acid profiles in a spontaneous mouse model of metabolic syndrome. *Scientific reports.* 2017;7(1):15876.
18. Müller M, Hernández MAG, Goossens GH, et al. Circulating but not faecal short-chain fatty acids are related to insulin sensitivity, lipolysis and GLP-1 concentrations in humans. *Scientific reports.* 2019;9(1):12515.
19. Sarah K, Nicolaas D, John T, Gabriella TH, Engelen M. Reduced Short-Chain Fatty Acid (SCFA) Plasma Concentrations Are Associated with Decreased Psychological Well-Being in Clinically Stable Congestive Heart Failure Patients. *Current Developments in Nutrition.* 2020;4(Supplement_2):42-42.
20. Cohen S, Kamarck T, Mermelstein R. A global measure of perceived stress. *J Health Soc Behav.* 1983;24(4):385-396.
21. Kroenke K, Spitzer RL. The PHQ-9: A new depression diagnostic and severity measure. *Psychiatric Annals.* 2002;32(9):509-515.
22. Corson K, Gerrity MS, Dobscha SK. Screening for depression and suicidality in a VA primary care setting: 2 items are better than 1 item. *Am J Manag Care.* 2004;10(11 Pt 2):839-845.
23. Kotronoulas GC, Papadopoulou CN, Papapetrou A, Patiraki E. Psychometric evaluation and feasibility of the Greek Pittsburgh Sleep Quality Index (GR-PSQI) in patients with cancer receiving chemotherapy. *Support Care Cancer.* 2011;19(11):1831-1840.
24. Beck SL, Schwartz AL, Towsley G, Dudley W, Barsevick A. Psychometric evaluation of the Pittsburgh Sleep Quality Index in cancer patients. *J Pain Symptom Manage.* 2004;27(2):140-148.
25. Carpenter JS, Andrykowski MA. Psychometric evaluation of the Pittsburgh Sleep Quality Index. *J Psychosom Res.* 1998;45(1):5-13.
26. Smets EM, Garssen B, Bonke B, De Haes JC. The Multidimensional Fatigue Inventory (MFI) psychometric qualities of an instrument to assess fatigue. *J Psychosom Res.* 1995;39(3):315-325.
27. National Cancer Institute. Patient-Reported Outcomes version of the Common Terminology Criteria for Adverse Events (PRO-CTCAE). http://outcomes.cancer.gov/tools/pro-ctcae.html. Published 2012 http://outcomes.cancer.gov/tools/pro-ctcae.html. Accessed.
28. Golden-Kreutz DM, Browne MW, Frierson GM, Andersen BL. Assessing stress in cancer patients: a second-order factor analysis model for the Perceived Stress Scale. *Assessment.* 2004;11(3):216-223.

© 2025 Xiao C et al. *JAMA Network Open*.

29. Xiao C, Beitler JJ, Higgins KA, et al. Associations among human papillomavirus, inflammation, and fatigue in patients with head and neck cancer. *Cancer.* 2018.
30. Xiao C, Beitler JJ, Peng G, et al. Epigenetic age acceleration, fatigue, and inflammation in patients undergoing radiation therapy for head and neck cancer: A longitudinal study. *Cancer.* 2021;127(18):3361-3371.

© 2025 Xiao C et al. *JAMA Network Open*.
