# Supplementary material for: Neighborhood Deprivation and Biological and Psychosocial Outcomes for Head and Neck Cancer
Source: JAMA Netw Open. 2025 Oct 21;8(10):e2538569. doi: 10.1001/jamanetworkopen.2025.38569 (PMC12541531; doi:10.1001/jamanetworkopen.2025.38569)
Supplement: Supplement 2. — Data Sharing Statement [file jamanetwopen-e2538569-s002.pdf]

Data Sharing Statement

Xiao. Neighborhood Deprivation and Biological and Psychosocial Outcomes for Head and Neck Cancer. *JAMA Netw Open*. Published October 21, 2025. doi:10.1001/jamanetworkopen.2025.38569

Data
Data available: No
